# EBUS-TBLC increase the diagnosis rate in different type of peripheral pulmonary lesions

**DOI:** 10.7150/jca.91169

**Published:** 2024-01-01

**Authors:** Ziwei Zhu, Jikai He, Yanyan Cao, Nana Wang, Xiaochen Xie, Guihong Wei, Haiyan Lin, Ying Chen, Suan Sun, Zili Meng, Wei Zhao, Paul Zarogoulidis, Panagoula Oikonomou, Christina Nikolaou, Charalampos Charalampidis, Haidong Huang, Wei Chen

**Affiliations:** 1 Department of Respiratory and Critical Care Medicine, The Huaian Clinical College of Xuzhou Medical University, Huai'an 223300, China.; 2 Research Center for the prevention and treatment of drug resistant microbial infecting, Youjiang Medical University for Nationalities, Baise 533000, China.; 3 Department of Pathology, the Affiliated Nanjing Hospital of Nanjing Medical University, Nanjing 210006, China.; 4 Department of Respiratory and Critical Care Medicine, The Affiliated Huaian No.1 People's Hospital of Nanjing Medical University, Huai'an 223300, China.; 5 Department of Pathology, The Affiliated Huaian No.1 People's Hospital of Nanjing Medical University, Huai'an 223300, China.; 6 Department of Respiratory and Critical Care Medicine, The First Affiliated Hospital of Naval Medical University, Shanghai, China.; 7 Pulmonary Department, Bioclinic Private Clinic, Aristotle University of Thessaloniki, Thessaloniki, Greece.; 8 2 ND Surgery Department, University General Hospital of Alexandroupolis, Democritus University of Thrace, Alexandroupolis, Greece.; 9 Pathology Department, University of Cyprus, Cyprus.

**Keywords:** EBUS, PPLs, type, eccentric, concentric.

## Abstract

**Background and objective:** Recently, endobronchial ultrasonography with guide sheath-guided (EBUS-GS) has been increasingly used in the diagnosis of peripheral pulmonary lesions (PPLs) from human natural orifice. However, the diagnostic rate is still largely dependent on the location of the lesion and the probe. Here, we reported a new procedure to improve the diagnostic rate of EBUS-transbronchial lung cryobiopsy (EBUS-TBLC), which performed under general anesthesia with laryngeal mask airway (LMA) in all of the patients. This study retrospectively evaluated the diagnosis of PPLs with 'blind-ending' type (Type I) and 'pass-through' type procedures (Type II) of EBUS-GS-TBLB or EBUS-TBLC respectively.

**Methods:** Retrospective review of 136 cases performed by EBUS-GS-TBLB or EBUS-TBLC for PPLs over 2 years.

**Results:** A total of 126 cases EBUS-GS-TBLB or EBUS-TBLC were performed during the study period. Among them, 66 (52.4%) were performed Type I and 60 (47.6%) were performed Type II. Clinical baseline characteristics did not differ between two groups. The overall diagnosis rate of 126 patients with EBUS-GS-TBLB or EBUS-TBLC was 73% (92/126), and different method type have significant influence on the diagnostic yield (*P* = 0.012, ***x*^2^** = 4.699). Among them, diagnostic yields for Type I with forceps biopsy (n=34), Type I with cryobiopsy (n=32), Type II with forceps biopsy (n=30), and Type II with cryobiopsy (n=30) were 72.5%, 64.5%, 70.4% and 74.2% respectively (Figure 2A). The study further compared the outcomes of different procedures in concentric and eccentric lesion. Diagnostic yields for Type I with eccentric (n=30), Type I with concentric (n=36), Type II with eccentric (n=34), and Type II with concentric (n=26) were 58.2%, 76.9%, 60.2% and 74.8%, respectively (*P* < 0.05). The incidence of complications in 126 patients was 2.6%.

**Conclusion:** EBUS-GS-TBLB and EBUS-TBLC both are very safe and highly diagnostic technique; different method types have significant influence on the diagnostic yield. Moreover, Type II procedure has higher diagnostic yield. In addition, Type I with eccentric had the lowest diagnosis yield.

## Introduction

Lung cancer is one of the common diseases of respiratory system, early screening and detection is the key to improve the overall prognosis of lung cancer.**1** Solid lung lesions are one of the common imaging manifestations of respiratory diseases. With the popularization of chest computer tomography (CT) technology, more and more lung lesions have been found, and the diagnosis of benign and malignant lesions is still based on pathological diagnosis.**2, 3**

Conventional bronchoscopy is difficult to find the peripheral pulmonary lesions (PPLs), and can do nothing for peripheral lung cancer.**4** Clinical development of early diagnosis of peripheral lung cancer is urgently needed. With the rapid development of interventional techniques for lung cancer, endobronchial ultrasound-guided transbronchial lung biopsy/cryobiopsy with a guided sheath (EBUS-GS-TBLB or EBUS-TBLC) has gradually matured.**5** The small ultrasound probe can enter the airway through the bronchoscope biopsy hole to perform a 360° cross-sectional scan of the lesion site, display the ultrasound image of the lesion and surrounding tissues, and explore the PPLs that cannot be observed by ordinary bronchoscope, which improves the positive rate of diagnosis of lung cancer in Chinese patients with low complication rate.**6-9**

Studies shown that cryobiopsy has been used to diagnose interstitial lung disease, lung cancer, PPLs, and as a post-transplant test.^10^, ^11^ The samples obtained by cryobiopsy meet the histopathological requirements, and have the advantages of large specimen, less false error, more alveolar tissue and high diagnostic rate.**12-15** Cryobiopsy is considered the first option for the diagnosis of benign lesions. However, the diagnostic rate is still largely dependent on the location of the lesion and the probe. Therefore, a new method was designed to improve the diagnostic rate of the technique by direct penetration of the probe into the lesion.

Our study aimed to evaluate the diagnosis of PPLs with blind-ending type (Type I) and pass-through type procedures (Type II) of EBUS-GS-TBLB or EBUS-TBLC. In addition, we would also evaluate and optimize the technique in combination with information on complications, clinical data and pathological diagnosis.

## Methods

### Study design

This study was conducted in the department of respiratory and critical care medicine, The Huai'an Clinical College of Xuzhou Medical University. The respiratory medicine unit performs ∼3000 respiratory endoscopies per year, including advanced diagnostic and therapeutic bronchoscopies. Patients with endobronchial lesions biopsied during initial airway examination, as well as patients with incomplete clinical data or information were excluded.

126 Patients with PPLs from Nov. 1. 2020 to Oct. 31. 2022 were enrolled. All patients were diagnosed by chest CT or PET-CT. There were 60 males and 66 females aged from 28 to 80 years. 62 patients had bronchial (diameter ≥1.4 mm, measured from CT images) penetration in the lesion, while 64 cases without bronchial penetration. For patients' lesions with eligible bronchial, we performed pass-through type (Type II) EBUS-GS-TBLB or EBUS-TBLC; for patients' lesions without eligible bronchial, we performed blind-ending type (Type I) EBUS-GS-TBLB or EBUS-TBLC.

#### Inclusion criteria

Patients with PPLs diagnosed by chest CT or PET-CT;

No history of extra-pulmonary malignant tumor;

Patients willing to cooperate.

#### Exclusion criteria

Lesions located in superior lobe of right lung and left superior division bronchus (for the cryoprobe can't reach this area);

Severe pulmonary infection with high fever, cardiopulmonary function is extremely poor;

Bronchial asthma attack period or active large hemoptysis patients;

Abnormal function of heart, lung, brain, kidney and other organs.

### EBUS-GS-TBLB and EBUS-TBLC procedure

#### EBUS-GS-TBLB

**Figure 1:**
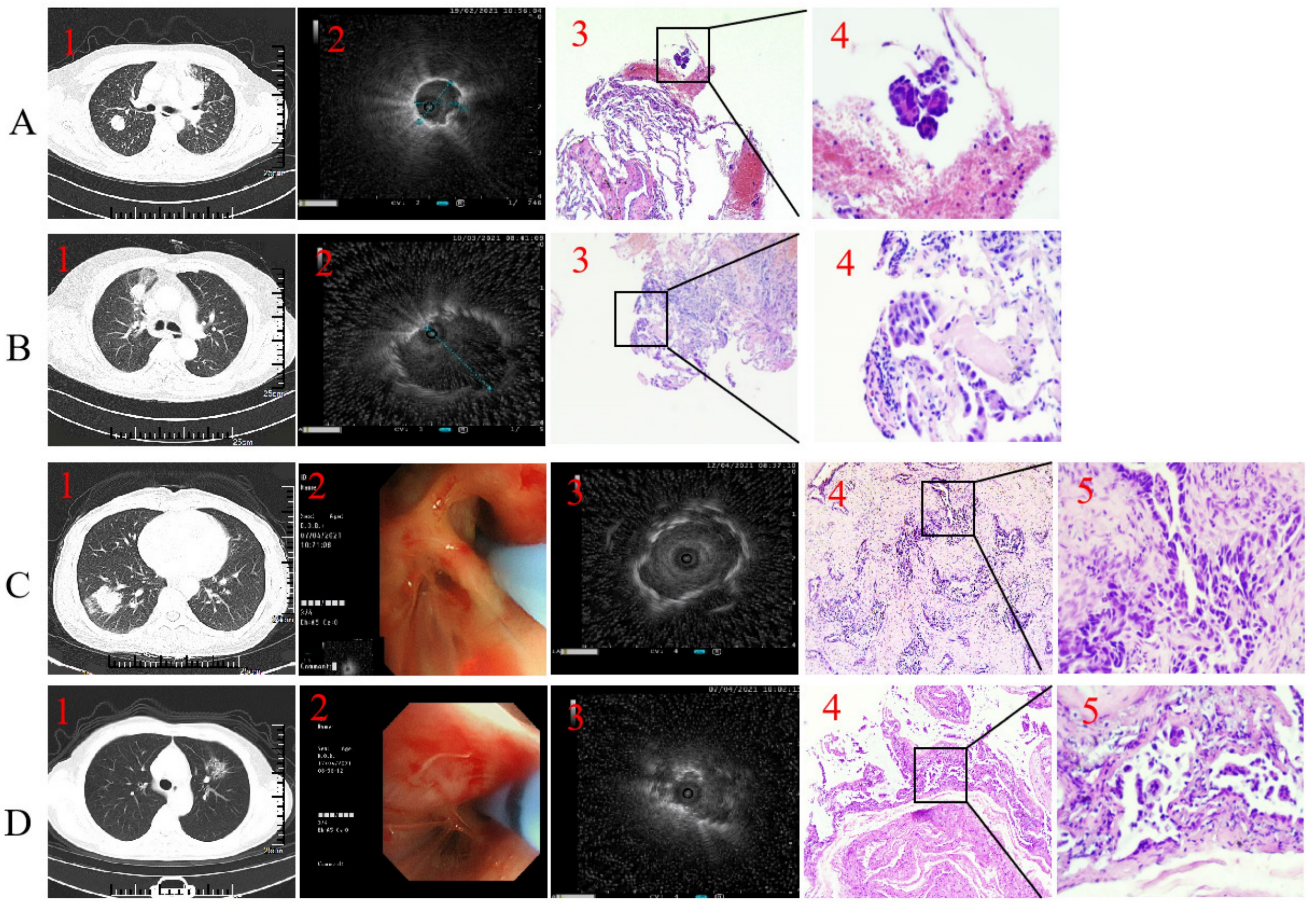
** Representative case.** Type I with forceps biopsy case. 1. Axial computed tomography of the chest demonstrated a 19.8×19.6 mm peripheral pulmonary lesion over right upper lobe and the distal of the passing bronchus in the lesion is blind-ending; 2. Radial endobronchial ultrasound revealed a concentrically orientated lesion; 3-4. Pathological diagnosis was adenocarcinoma by forceps biopsy (H&E, 3 was 100-fold magnification; 4 was 400-fold magnification).** B.** Type II with forceps biopsy case. 1. Axial computed tomography of the chest demonstrated a 22.5×17.3 mm peripheral pulmonary lesion over right upper lobe and bronchus passing through the lesion; 2. Radial endobronchial ultrasound revealed a eccentrically orientated lesion; 3-4. Pathological diagnosis was adenocarcinoma by forceps biopsy (H&E, 3 was 100-fold magnification; 4 was 400-fold magnification).** C.** Type I with cryobiopsy case. 1. Axial computed tomography of the chest demonstrated a 28.3×27.6 mm peripheral pulmonary lesion over right low lobe and the distal of the passing bronchus in the lesion is blind-ending; 2. A 1.9 mm cryoprobe with a prophylactic blocking balloon were placed at the posterior opening of the right lower lung; 3. Radial endobronchial ultrasound revealed a concentrically orientated lesion; 4-5. Pathological diagnosis was adenocarcinoma by cryobiopsy (H&E, 4 was 100-fold magnification; 5 was 400-fold magnification).** D.** Type II with cryobiopsy case. 1. Axial computed tomography of the chest demonstrated a 27.1×26 mm peripheral pulmonary lesion over left upper lobe and bronchus passing through the lesion; 2. A 1.9 mm cryoprobe with a prophylactic blocking balloon were placed at the posterior opening of the left upper lobe; 3. Radial endobronchial ultrasound revealed a eccentrically orientated lesion; 4-5. Pathological diagnosis was adenocarcinoma by cryobiopsy (H&E, 4 was 100-fold magnification; 5 was 400-fold magnification).

**Figure 2:**
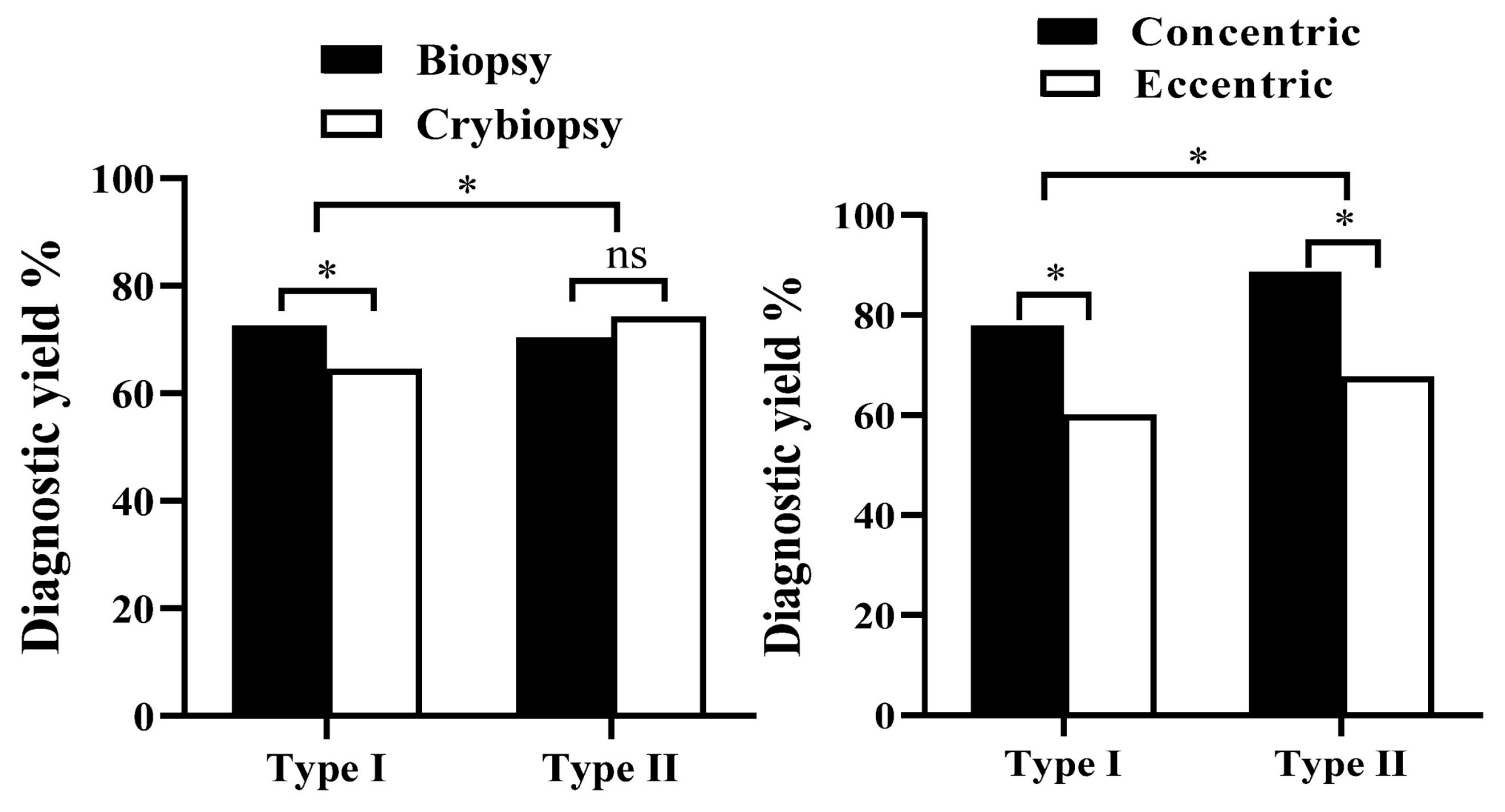
** The diagnostic yield of the Type I *versus* Type II between different groups.** * P<0.05.

EBUS-GS was performed using the standard techniques as previously reported**.^16^** A representative case of EBUS-GS in a patient with PPLs is shown in Fig. 1. Briefly, using a thin-section chest CT scan for guidance, a thin bronchoscope (BF-P260F; Olympus, Tokyo, Japan) was advanced as close as possible to the target peripheral lesion under general anesthesia with laryngeal mask airway (LMA). Then, a 20MHz radial EBUS probe (UM-S20-17S; Olympus), covered with a GS (K-201; Olympus) was introduced through the working channel of the bronchoscope to precisely locate the target lung lesion. According to previous studies,^16^-^19^ radial probe EBUS findings of the target peripheral lesion were classified as within, adjacent to, or outside of the lesion (Fig. 2). After identifying the target lesion on the radial probe EBUS, the guide sheath was locked in place and R-EBUS probe remove, then subsequent forceps biopsy and brush cytology were performed. Five to eight biopsies were taken during each round of the procedure.**5** Biopsied specimens were fixed in formalin solution and sent to pathology lab immediately for processing and analysis.

#### EBUS-TBLC

Patients were performed transbronchial lung cryobiopsy (TBLC) under LMA (Well lead Medical Co., Ltd, Guangzhou, China). We performed TBLC with a flexible bronchoscope (5.9 mm distal end diameter, 2.8 mm working channel diameter; EVIS BF-1T260, Olympus, Tokyo, Japan) and a 1.9 mm cryoprobe (ERBECRYO 2; Erbe Elektromedizin GmbH, Tubigen, Germany). A 1.4 mm 20-MHz radical probe (UM-S20-17S; Olympus, Tokyo, Japan) was used to identify a target legion in the peripheral pulmonary region and measured the depth of the lesions at the same time. The dilation balloon (BDC-10/55-7/18; Micro-Tech (Nanjing) Co., Ltd, Nanjing, China) was routinely used to achieve hemostasis. A disposable biopsy forceps was used to clamp the front end of the dilation balloon from the apex of the bronchoscope, and then the balloon was placed in the segmental or subsegmental bronchus.

After identifying the target lesion, the cryoprobe was inserted via the working channel of the bronchoscope and was placed at the desired location under direct visualization on bronchoscopy. The lesion was frozen for 4-6 seconds using a cryoprobe. In order to alleviate the damage of the cryoprobe to the mucosa and vocal cords, The bronchoscope was then immediately removed with the cryoprobe along with the tissues, accompanied by the release of footswitch. The dilation balloon was then inflated for 2 minutes immediately after removing the bronchoscope. After the bronchoscope was reinserted to assess for hemostasis, TBLC was repeated for 3-5 times until a sample with adequate volume was obtained. The tissue samples were immediately fixed in 10% neutral-buffered formalin.

### Postoperative management

All patients were admitted to the resuscitation room postoperatively and underwent chest X-ray or CT scan exams within 3 hours, and those who received cryobiopsy were carefully examined airway mucosa, vocal cord structure, and range of motion following the procedure to evaluate for the cryobiopsy-related complication.

### Statistical analysis

We included all eligible patients from the study opening to closing dates in our analyses. Statistical results were analyzed in a double-blind manner with patient clinical treatment information. For variables assumed to be normally distributed, data are expressed as mean ± SD, whereas for variables non-normally distributed, data are expressed as median. Categorical data are expressed in absolute numbers and percentages. Statistical software SPSS 23 was used in this study. The significance of distribution differences between groups was estimated by *χ*^²^ test or Fisher's exact test, and all statistical tests were two-sided probability tests. The independent sample t test was used to compare two baseline data groups. Using *P* < 0.05 was considered statistically significant.

## Results

### Clinical baseline characteristics

A total of 136 cases were performed by EBUS-GS-TBLB or EBUS-TBLC procedures. 10 cases were excluded: pathological information was missing in 3 cases, localization failed in 7 cases. Total 126 cases were included for analysis. Representative samples cases were presented in Figure 1.

Among them, 66 (52.4%) were performed Type I and 60 (47.6%) were performed Type II. 22 (17.5%) cases had lesions in the middle/ lingular lobe, and 104 (82.5%) cases in the lower lobe. The mean diameter of the lesion was 28.21 mm. 62 (49.2%) cases of the lesions were concentric with the probe, while 64 (50.8%) cases of the lesions were eccentric with the probe. In concentric group, 36 (58%) were performed Type I, and 26 (42%) were performed Type II; In eccentric group, 30 (46.9%) were performed Type I, and 34 (53.1%) were performed Type II. In forceps biopsy, 34 (53.1%) cases were performed Type I, and 30 (46.9%) cases were performed Type II. In cryobiopsy, 32 (51.6%) cases were performed Type I, and 30 (48.4%) cases were performed Type II. Clinical baseline characteristics did not differ between the forceps biopsy and cryobiopsy groups (Table 1).

### Procedure characteristics

Type I and Type II procedures were randomly selected forceps biopsy and cryobiopsy to obtain local tissue specimen. The median procedure time for all enrolled patients was 37.2 min (20-50 min). The duration time of Type II procedure was slightly shorter than the Type I, but there was no statistical significance (*P*=0.286). Details of procedures are listed in Table 1.

### Comparison of accuracy of pathological diagnosis between different procedures

The overall diagnosis rate of 126 patients with EBUS-GS-TBLB OR EBUS-TBLC was 73% (92/126). In terms of the influence of factors on the diagnosis yield, we found that the diagnostic yield in Type II (46/60, 76.7%) higher than in Type I (46/66, 69.7%), and different method type have significant influence on the diagnostic yield (*P* = 0.012, ***x*^2^** = 4.699) (Table 2). The study compared the outcomes of different procedures in forceps biopsy and cryobiopsy. Diagnostic yields for Type I with forceps biopsy (n=34), Type I with cryobiopsy (n=32), Type II with forceps biopsy (n=30), and Type II with cryobiopsy (n=30) were 72.5%, 64.5%, 70.4% and 74.2% respectively (Figure 2A).

The study further compared the outcomes of different procedures in concentric and eccentric lesion. Diagnostic yields for Type I with eccentric (n=30), Type I with concentric (n=36), Type II with eccentric (n=34), and Type II with concentric (n=26) were 60%, 77.8%, 67.6% and 88.5%, respectively (*P* < 0.05) (Figure 2B).

### Comparison of pathological diagnosis between different procedures

For Type I with eccentric group (n=30), among which 6 cases were diagnosed as malignant (3 adenocarcinoma, 2 squamous cell carcinoma, and 1 metastatic carcinoma), 6 cases were undiagnosed as malignant but these cases were diagnosed as malignant by surgical operation or CT-guided lung puncture biopsy, 12 cases were diagnosed as benign, and 5 cases were not definitive diagnosed as benign disease.

For Type I with concentric group (n=36), pathological diagnosis was obtained in 28 cases, among which 19 cases were diagnosed as malignant (10 adenocarcinoma, 5 squamous cell carcinoma, 1 small cell carcinoma, 1 unclassified carcinoma, and 1 metastatic carcinoma), 4 case was undiagnosed as malignant but diagnosed as malignant by surgical operation or CT-guided lung puncture biopsy, 9 cases were diagnosed as benign, and 3 cases were not diagnosed as benign.

For Type II with eccentric group (n=34), pathological diagnosis was obtained in 23 cases, among which 14 cases were diagnosed as malignant (8 adenocarcinoma, 2 squamous cell carcinoma, 3 unclassified carcinoma, and 1 metastatic carcinoma), 5 cases were undiagnosed as malignant but diagnosed as malignant by surgical operation or CT-guided lung puncture biopsy, 9 cases were diagnosed as benign, and 5 case was not diagnosed as benign.

For Type II with concentric group (n=26), pathological diagnosis was obtained in 23 cases, among which 17 cases were diagnosed as malignant (8 adenocarcinoma, 4 squamous cell carcinoma, 1 small cell carcinoma, 1 mucinous carcinoma, and 3 unclassified carcinoma), 0 cases were undiagnosed as malignant (cancerous tissue was visible but tumor type was not clear), 6 cases were diagnosed as benign, and 3 cases were not definitive diagnosed as benign.

The details of the histological findings are displayed in Table 3.

### Comparison of complication rate in different groups

A total of 126 patients underwent EBUS-GS-TBLB or EBUS-TBLC and were well tolerated. 1 case showed small amount of bleeding at the puncture site in Type I group, 1 case in Type II group. All patients had hemostasis after suction with local 4℃ saline or adrenaline instillation. Only one hypoxemia occurred in each Type I group and Type II group. All patients were given elevated oxygen concentration and returned to the ward after the operation was stopped. There was no statistically significant difference between the two groups. Moreover; there was no statistically significant difference between the two groups regarding adverse effects like hemorrhage.

Especially, no patient who performed TBLC had damage of mucosal or vocal cord.

## Discussion

Recent years, the equipment and technology of endobronchial ultrasound have been continuously improved, and the diagnostic rate of transbronchial biopsy has been continuously improved. According to reported, the positive rate of EBUS-GS-TBLB or EBUS-TBLC for PPL was 58.82%-79.29%.**5** With this technique, the bronchoscope was sent to the distal bronchus of the segment where the concentrated lesions were displayed by chest CT, and the radial probe was inserted into the guide sheath and sent along the biopsy passage for ultrasonic exploration**.^20^, ^21^** After finding the best image of lesions, the guide sheath was fixed, and the probe was extracted for biopsy. The fixation of the guide sheath at the lesion facilitates multiple sampling, increases diagnostic rates and reduces the risk of bleeding.**22, 23** EBUS-GS-TBLB or EBUS-TBLC technology solves the technical problem that it is difficult for conventional bronchoscopy to reach small peripheral airways, and the ultrasonic probe improves the positive rate of PPLs detected by bronchoscopy. Literature shows that the diagnostic accuracy of EBUS-GS-TBLB for PPLs is between 68.9%-87.5%,**24** while our diagnostic yield reached to 73% (92/126) with a low complication rate of 2.6% through EBUS-GS-TBLB or EBUS-TBLC. In the study, we divided all the enrolled cases into Type I and Type II, and the analysis found the diagnostic yield in Type II (46/60, 76.7%) higher than in Type I (46/66, 69.7%), and different method type have significant influence on the diagnostic yield (*P* = 0.012, ***x*^2^** = 4.699).

In 2008, Dr. Hetzel first proposed the probability of cryogenic biopsy, and reported the diagnosis of 12 cases of endobronchial tumor by cryogenic technique for the first time.**10** It was found that the soft bronchoscopy specimen collected at low temperature not only maintained high histological integrity, but also preserved its internal molecular markers. Cryobiopsy is a biopsy method in which ice crystals adhere to the lung tissue by applying refrigerant to the tip of the frozen probe, and the adhered lung tissue is removed through the bronchus.

In this study, our enrolled cases included 64 cases forceps biopsy and 62 cases cryobiopsy, and the diagnostic yield for the two groups were 70.3% and 75.6%, and it was not statistically significant. We speculate that it might be due to the small number of cryobiopsy cases, and we will further increase the number of cases in the future to further observe the influence of biopsy method on the pathological diagnosis of different method types of patients.

Kho et al. demonstrated the orientation remained an important factor affecting diagnostic yield, and cryobiopsy indeed significantly increased the diagnostic yield of eccentrically and adjacently orientated lesions.**5** For further analyze the influence of different method type on the diagnosis yield, we divided 126 cases into four groups, including Type I with eccentric group (n=30), Type I with concentric group (n=36), Type II eccentric group (n=34), Type II with concentric group (n=26). It showed that Type I with eccentric (60%) had the lowest diagnosis yield, and this is mainly due to the location of the lesion.

Our study suggests that Type II procedure has higher diagnostic yield and different subtype (concentric or eccentric) have significant influence on the diagnostic yield, Type II with concentric has a higher diagnosis rate than eccentric. But different biopsy methods (forceps biopsy or cryobiopsy) did not determine the final diagnosis. In addition, Type I with eccentric had the lowest diagnosis yield. Interestingly, we found that forceps biopsy is more accuracy than cryobiopsy in Type I. Multi-center randomized controlled trials are needed to further verify the results of this study. Finally, additional navigation methods, such as robotic bronchoscopy or cone beam-CT certainly can enhance the diagnostic result.

## Figures and Tables

**Table 1:** Comparison of clinical characteristic condition between different procedures

	Overall	Method type		*P value*
	EBUS-GS-TBLB OR EBUS-TBLC	Type I	Type II	
**Subjects**	126	66 (52.4)	60 (47.6)	
**Patient characteristic**				
**Age**				0.116
≥60	46 (36.5)	20 (43.5)	26 (56.5)	
<60	80 (63.5)	39 (48.8)	41 (51.2)	
**Sex**				0.188
Male	60 (47.6)	32 (45.6)	28 (54.4)	
Female	66 (52.4)	30 (45.5)	36 (54.5)	
**Lesion location**				0.274
Middle/ Lingular lobe	22 (17.5)	10 (45.5)	12 (54.5)	
Lower lobe	104 (82.5)	58 (55.8)	46 (44.2)	
**Leision size**				0.228
< 20mm	37 (29.4)	20 (54.1)	17 (45.9)	
20-30 mm	89 (70.6)	38 (42.7)	51 (57.3)	
**Smoking history**				1.000
Smoking	74 (58.7)	34 (45.9)	40 (54.1)	
no smoking	52 (41.3)	32 (61.5)	20 (38.5)	
**Orientation**				0.659
Eccentric	64 (50.8)	30 (46.9)	34 (53.1)	
Concentric	62 (49.2)	36 (58.1)	26 (41.9)	
**Biopsy method**				0.106
Forceps biopsy	64 (50.8)	34 (53.1)	30 (46.9)	
Cryobiopsy	62 (49.2)	32 (51.6)	30 (48.4)	
**Procedure characteristics**				
Time min	37.2 (20-50)	39.0 (26-56)	35.3 (20-50)	0.286

**Table 2:** Comparison of pathological diagnosis between different procedures

	Diagnostic yield	*P* value	*x* ^2^
**Subjects**			
**Method type**		**0.012**	**4.699**
Type I	46/66 (69.7)		
Type II	46/60 (76.7)		
**Biopsy method**		0.420	0.048
Forceps biopsy	45/64 (70.3)		
Cryobiopsy	47/62 (75.6)		
**Lesion location**		1.015	10.37
Middle/Lingular lobe	16/22 (72.7)		
Lower lobe	76/104 (73.1)		
**Lesion size**		1.425	3.628
< 20 mm	26/37 (70.3)		
20-30 mm	66/89 (74.2)		
**Orientation**		**0.004**	**6.658**
Eccentric	42/64 (65.6)		
Concentric	50/62 (80.6)		

**Table 3:** Pathological histologists diagnosis of biopsy in different groups

	Overall	Type I		Type II	
	EBUS-GS-TBLB and EBUS-TBLC	Eccentric	Concentric	Eccentric	Concentric
	Diagnostic cases / All cases (diagnostics yield)				
**Conclusive histology**	92/126 (73)	18/30 (60)	28/36 (77.8)	23/34 (67.6)	23/26 (88.5)
**Malignant**					
Adenocarcinoma carcinoma	29/38 (76.3)	3/6 (50)	10/14 (71.4)	8/10 (80)	8/8 (100.0)
Squamous cell carcinoma	13/16 (81.3)	2/3 (66.7)	5/5 (100)	2/3 (66.7)	4/4 (100.0)
Adenosquamous carcinoma	0/1 (00.0)	0/0 (0)	0/0 (0)	0/1 (00.0)	0/0 (0)
small cell carcinoma	3/4 (75)	0/1 (100.0)	2/2 (100)	0/0 (0)	1/1 (100.0)
mucinous carcinoma	1/1 (100.0)	0/0 (0)	0/0 (0)	0/0 (0)	1/1 (100.0)
Unclassified carcinoma	7/9 (77.8)	0/1 (0)	1/1 (100)	3/4 (75)	3/3 (100.0)
metastatic carcinoma	3/4 (75.0)	1/1 (100.0)	1/1 (100)	1/2 (50)	0/0 (0)
**benign lesions**					
Tuberculosis	7/8 (87.5)	1/1 (100.0)	2/2 (100.0)	3/3 (100.0)	1/2 (50.0)
Granulomatous inflammation	2/2 (100.0)	0/0 (0)	0/0 (0)	1/1 (100.0)	1/1 (100.0)
inflammation	15/18 (83.3)	8/9 (88.9)	4/6 (66.7)	2/2 (100)	1/2 (50)
interstitial pneumonia	3/6 (50)	0/2 (00.0)	2/2 (100)	1/1 (100)	0/1 (0)
Hyperplasia of interstitial	3/7 (42.9)	1/2 (50)	0/1 (00.0)	1/3 (33.3)	1/1 (100)
Sjogren's syndrome interpulmonary degeneration	1/2 (50.0)	1/1 (100.0)	0/0 (0)	0/1 (00.0)	0/0 (00.0)
**Inconclusive histology**					
**Nondiagnostic**					
gland	2/6 (33.3)	1/2 (50.0)	1/2 (50)	0/2(0)	0/0 (0)
Necrotic tissue	1/1 (100.0)	0/0 (0)	0/0 (0)	0/0 (0)	1/1 (100.0)
Bronchial epithelium	1/2 (50.0)	0/1 (00.0)	0/0 (0)	0/0 (0)	1/1 (100.0)
Haemorrhagic lung tissue	1/1 (100.0)	0/0 (0)	0/0 (0)	1/1 (100)	0/0 (0)

## Abbreviations

CT: chest computer tomography
EBUS: endobronchial ultrasound
GS: guided sheath
PPLs: peripheral pulmonary lesions
TBLB/TBLC: transbronchial lung biopsy/cryobiopsy
